# Overview of waste bank application in Indonesian regencies

**DOI:** 10.1177/0734242X241242697

**Published:** 2024-04-10

**Authors:** Arie Budiyarto, Beverley Clarke, Kirstin Ross

**Affiliations:** 1 College of Science and Engineering, Flinders University, Bedford Park, SA, Australia; 2 College of Humanities, Arts and Social Sciences, Flinders University, Bedford Park, SA, Australia

**Keywords:** Municipal solid waste, municipal solid waste management, waste banks

## Abstract

Managing municipal solid waste (MSW) is a critical for Indonesia, as the country produces a substantial amount of waste annually. However, Indonesia’s recycling rate remains limited, less than 25% of its waste, with the rest ending up in landfills. To address this, waste banks have emerged as a community-based solution to enhance MSW management through recycling. Although waste banks currently contribute only 7% to recyclable waste management, they hold promising potential, especially considering their close ties to households, the primary waste producers in Indonesia. Unfortunately, documentation of waste bank successes in Indonesian regencies is scarce, as most success stories are limited to major cities. This article conducts a literature review on waste bank implementations across various regencies, evaluating their accomplishments, obstacles and potential contributions to local MSW management. The review draws upon scholarly publications and various government reports, regulations and websites dedicated to updates on waste bank activities. Waste banks play a crucial role in enhancing environmental quality by promoting proper waste disposal and reducing landfill waste. They create economic opportunities, increasing income for both customers and administrators. Additional services, such as banking facilities encompassing savings, loans, daily necessities and bill payments, amplify their significance. To fully harness the potential of waste banks, support is imperative. Establishing adequate infrastructure and providing capacity-building for administrators are essential. Although regulatory frameworks offer opportunities, the impact of regency-level regulations on waste bank growth varies and necessitates further examination. Support mechanisms should be tailored to align with local characteristics and requirements.

## Highlights

- Regencies in Indonesia face challenges in providing municipal solid waste (MSW) management facilities.
- Waste banks offer a community-based solution for effective MSW management.
- Waste banks show potential in enhancing MSW management effectiveness.

## Introduction

Solid waste management is a pressing environmental issue faced by many developing countries, including Indonesia (Abubakar et al., 2022; Ferronato and Torretta, 2019; Khan et al., 2022a; Rodić and Wilson, 2017; Zohoori and Ghani, 2017). On one side, Indonesia generates a vast amount of waste every year, reaching 68.5 million tonnes in 2022 (Ministry of Environment and Forestry Republic of Indonesia, 2023a), nearly equivalent to the waste generated by developed countries like Australia, which amounted to 75.8 million tonnes in 2018–2019 (ABS, 2020). On the other hand, waste management in Indonesia remains severely inadequate. The average coverage of waste collection areas in Indonesia is only around 50% of the municipality’s total area (Ministry of Environment and Forestry Republic of Indonesia, 2023a; Mohammad Debby Rizani et al., 2016; Wibisono et al., 2020). This problem has arisen due to inadequate waste collection and transportation infrastructure, a consequence of limited operational funds allocated for waste management activities (Ramadhani and Aisyah, 2022; Zainal et al., 2021). Consequently, the insufficient coverage of waste collection results in a significant amount of unmanaged waste. This, in turn, leads to environmentally unfriendly practices such as dumping waste in rivers and burning waste (Aboyitungiye and Gravitiani, 2021; Simangunsong and Fajarwati, 2018). Another challenge faced by local governments is the poor quality of landfill waste management systems, particularly the ineffective operation of sanitary landfills (Artiningsih et al., 2019; Nursetiawan et al., 2021).

If the issue of solid waste management across Indonesia remains unaddressed, it poses significant ongoing and increasing risks to human health and the environment. Inadequate solid waste management can lead to environmental pollution and increased disease transmission (Hoornweg and Bhada-Tata, 2012; Khan et al., 2022b; Serge Kubanza and Simatele, 2020). Inappropriate waste management practices such as open burning and improper landfill applications also contribute to global warming (Ngusale et al., 2021; Siddiqua et al., 2022; Talang and Sirivithayapakorn, 2021). Waste disposal in waterways and rivers results in the pollution of water bodies and the accumulation of garbage, especially plastics, wood and bulky items, which can lead to flooding due to blocked water distribution channel (Vollmer and Grêt-Regamey, 2013).

The central government has been working on improving waste management performance in Indonesia, including promoting public–private partnership (PPP) schemes (Setya, 2023). PPP is considered a promising approach as it can alleviate the burden on local governments by fostering collaboration between them and business entities in providing waste management infrastructure and services (Sutana, 2023). However, the implementation of PPP in waste management has faced challenges and has not been fully developed, primarily due to complexities in regulations, technological requirements and financing credit schemes. By the end of 2021, only one waste management activity in Surabaya, East Java Province, the Benowo Waste Processing and Final Processing Site (TPPAS Benowo), utilized the PPP scheme (Coordinating Ministry for Economic Affairs of the Republic of Indonesia, 2021; Ministry of Home Affairs of the Republic of Indonesia, 2018). Therefore, alternative waste management approaches that are easy to implement, especially in regencies, are needed.

The Republic of Indonesia is divided into provinces, and provinces are further divided into regencies and cities. Each of these administrative units has its own regional government, with the governor leading the provincial government, the regent leading the regency government and the mayor leading the city government (The Government of the Republic of Indonesia, 2014). Marfiana and Kurniasih (2013) provided an overview of the general characteristics that differentiate regencies from cities. Regencies typically have larger land areas but lower population density and gross regional domestic income compared to cities.

**Figure 1.**
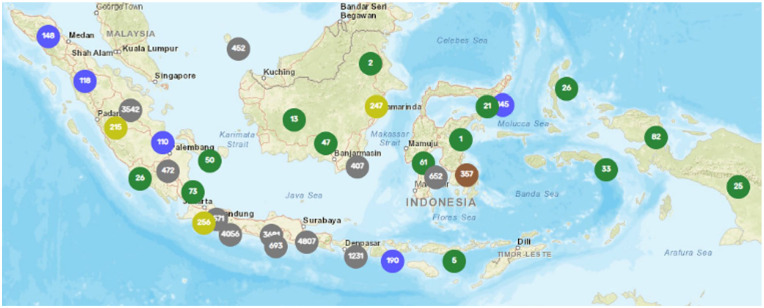
Distribution of waste banks across various provinces in Indonesia (taken from https://simba.menlhk.go.id/portal/#peta on 8 October 2023).

Based on Law 23/2014 related to local government (The Government of the Republic of Indonesia, 2014), the responsibility for waste management in each regency lies with its respective government. However, due to limited development funds commonly faced by regency governments in Indonesia (Hasan and Ridwan, 2021), waste management activities, particularly waste collection, have become a significant challenge. This is evident in cases such as Karimun Regency in Riau Islands Province (Tofani et al., 2022), Rokan Hulu Regency in Riau Province (Harahap, 2018) and Bone Bolango Regency in Gorontalo Province (Abdussamad et al., 2022) where the lack of waste infrastructure is a serious problem (Figure 1).

One approach that has been rapidly growing in Indonesia is community-based solid waste management through the establishment of waste banks. Since the first Waste bank was implemented in 2008 by Bambang Suwerda, an environmental practitioner and lecturer in the Badegan Bantul Regency of Indonesia, Special Province of Yogyakarta (Astuti et al., 2015), this innovation has been embraced extensively throughout the country. As of the end of 2022, there were 4341 waste banks distributed across 34 of 38 provinces, with an average of 10–11 Waste bank locations in each city or regency (Ministry of Environment and Forestry Republic of Indonesia, 2023a).

The success of the widespread establishment and increase in the number of waste banks in Indonesia can be attributed to the simple requirements for formation and operation (Wijayanti and Suryani, 2015). Waste banks do not require large land areas, complex equipment and can operate within neighbourhoods (Padawangi et al., 2016). As stated in the Ministry of Environment and Forestry of the Republic of Indonesia Regulation 14/2021, article 11

The primary consideration for selecting a waste bank location is its easy accessibility. In addition, the chosen site must meet minimum infrastructure requirements, including the presence of administrative offices, designated areas for waste sorting, waste collection points, waste storage facilities, and the availability of specific operational equipment.

Before the waste management activities by the newly established waste banks, which began in 2008, households typically did not sort their waste and disposed of it mixed with regular garbage or buried it in their backyard or discarded it in random places or undertook limited sorting of their waste and then sold it to waste pickers (Damanhuri, 2012; Damanhuri et al., 2014)

Without disregarding organic waste, which constitutes the largest portion of waste generated in Indonesia and its management remains a challenge, recyclable solid waste risen as one of the waste management challenge in Indonesia. Data released by the Ministry of Environment and Forestry of the Republic of Indonesia for the year 2022 shows that the quantity of recyclable solid waste is significant, approximately 26 million tonnes year^−1^, equivalent to around 38% of the total annual waste generated. This positions recyclable solid waste as the second-highest waste category by percentage, just below organic waste (55%). Out of the total 26 million tonnes of recyclable waste, only 22% have undergone recycling activities, whereas the rest have been disposed of in landfills. So, there is clear that management of recyclable waste in Indonesia is still insufficient (Ministry of Environment and Forestry Republic of Indonesia, 2023b).

Waste bank has an important role in the recycle waste management. Although the total amount of waste recycled in waste banks in a year contributes to less than 10% of the national recycling efforts (Ministry of Environment and Forestry Republic of Indonesia, 2023b), the presence of waste banks can encourage improvements in recyclable waste management specifically by the households (Maryati et al., 2018; Zakianis and Djaja, 2017). That role is very important since most of waste bank’s customers are households. Although there are currently no data available from the central or regional governments at the province/city/regency levels regarding the number of households becoming waste bank customers each year since 2008, the Ministry of Environment and Forestry of the Republic of Indonesia released data in mid-2023 indicating that waste banks across Indonesia have attracted the interest of around 350 thousands households in actively participating in waste sorting activities through waste banks and households are the biggest waste producers in Indonesia (Ministry of Environment and Forestry Republic of Indonesia, 2023b).

However, this significant role performed by waste banks has to date been performed in large cities only. For example, waste banks are located in in Medan in North Sumatera Province, Jakarta (Indonesia’s capital city), Bandung in West Java Province, Surabaya in East Java Province, Yogyakarta in Special Province of Yogyakarta, Banjarmasin in South Kalimantan Province, Balikpapan in East Kalimantan Province, Manado in North Sulawesi Province, Makassar in South Sulawesi Province and Denpasar in Bali Province, they can manage around 0.9 tonne of municipal solid waste (MSW) month^−1^ (Utami, 2013). A similar success is also evident with the ‘Malang’ waste bank in Malang City, East Java Province, which is even capable of managing 2.5 tonnes of MSW day^−1^ (Suryani, 2014). Little has been documented about success of waste banks in regencies. Therefore, more studies are warranted to investigate the role and performance of waste banks in improving the quality of waste management in other regencies of Indonesia.

The aim of this article is to examine the achievements and challenges of community-based waste management through waste bank programmes and to assess their potential in supporting local MSW management in Indonesian regencies. This article will contribute to the development of waste banks in Indonesian regencies, particularly in enhancing the management of recyclable waste generated by households.

## Methodology

A comprehensive literature search was conducted using the Scopus (https://www.scopus.com/search/form.uri?display=basic#basic) and Web of Science (https://www.webofscience.com/wos/woscc/basic-search) databases to gather relevant information. By using the keywords ‘waste bank’ AND characteristics OR Challenge OR Success in the Scopus database, resulting in 38 articles. Afterwards, a search with the same keywords was performed in the Web of Science database, but it yielded too many articles, specifically 1,543,859 articles, so the search results were not used. Therefore, for the Web of Science database, the keywords were changed to a more general ‘waste bank’, resulting in 72 articles. Thus, a total of 110 articles were obtained from the Scopus and Web of Science databases.

Following that, duplicate journals obtained from Scopus and Web of Science were checked using the EndNote referencing programme, resulting in the identification of 8 duplicated journals out of 110 articles, leaving a total of 102 articles. The next step involved skimming the titles with the keyword ‘waste bank’, resulting in 41 articles. This was followed by screening abstracts and research outcomes within the context of waste management through waste banks, specifically in Indonesia, resulting in 40 articles in total. The analysis of ‘waste bank characteristics, success stories, and challenges’ was conducted on the selected articles as part of a comprehensive review of waste banks in Indonesian regencies.

To gather additional information regarding ‘waste bank characteristics, success stories, and challenges in Indonesia’, snowball sampling was also conducted on articles available on the Google Scholar search engine, resulting in 66 relevant articles.

**Figure 2.**
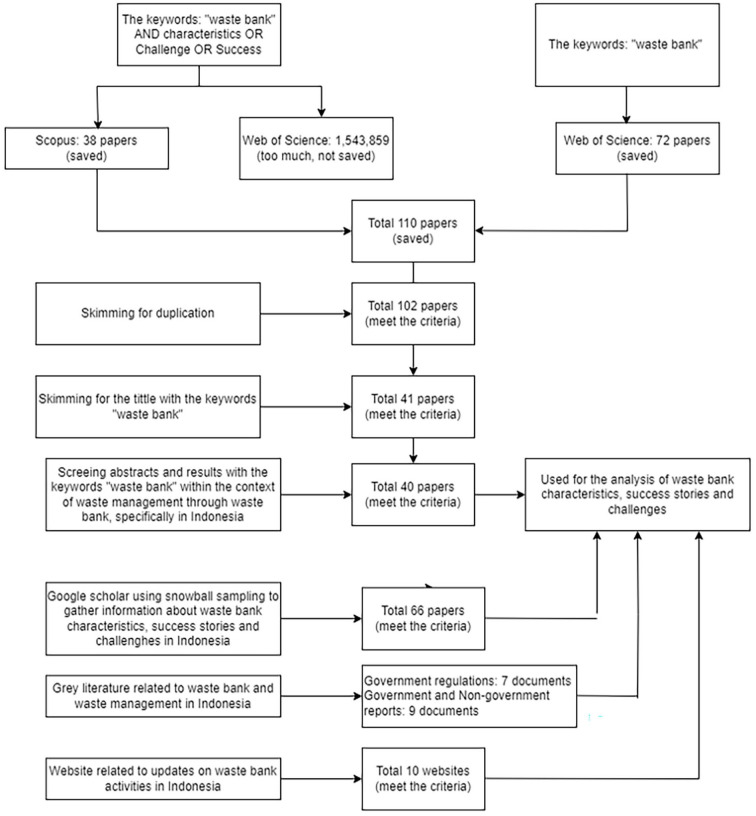
Literature search flowchart.

In addition, an analysis was conducted on grey literature, including regulations (seven documents) and reports (nine documents) issued by the government related to waste management in waste banks. To complement data related to the updates on waste bank activities in Indonesia, data analysis was also carried out on information obtained from 10 websites (Figure 2).

## What are waste banks?

A waste bank is a community-based waste management initiative that involve active participation from the community in waste sorting and recycling activities (Jaya and Machdum, 2023). The formal definition of a waste bank according to the regulations of the Minister of Environment and Forestry of the Republic of Indonesia number 14 of 2021 is as follow:

Waste Bank is a facility for managing waste with the principles of 3R (reduce, reuse, and recycle). It serves as an educational tool, encourages behavior change in waste management, and facilitates the implementation of Circular Economy. It is established and managed by the community, businesses, and/or local government.

The regulatory framework for waste bank operations is established through Regulation Number 14 of 2021, issued by the Minister of Environment and Forestry of the Republic of Indonesia (‘*Permenlhk 14/2021*’) (Ministry of Environment and Forestry Republic of Indonesia, 2021).

Based on the *Permenlhk 14/2021* (Ministry of Environment and Forestry Republic of Indonesia, 2021), there are two types of waste banks: central waste banks and unit waste banks. The characteristics of these types are outlined in Table 1.

**Table 1.** Characteristics of both central and unit waste bank.

	Central waste bank	Unit waste bank
Organizational form	Privately owned enterprises (Nasution, 2022) Privately owned enterprises managed by NGOs (Yayasan Bina Bhakti Lingkungan, 2022) As a part of technical operational unit within a government agency of the Regency (Iftitah and Musta’in, 2018) Government sponsored (Adventa et al., 2020) Village-owned enterprises (Luqmania et al., 2022)	CBO (Purwanto, 2019) Village-owned enterprises (Nasution and Agustin, 2020)
Coverage area	One central waste bank has a minimum coverage area that includes one village (Ministry of Environment and Forestry Republic of Indonesia, 2021) A village is an administrative area led by a village head and is typically comprised of around 500 or more families (Government of Republic of Indonesia, 2014)	One unit waste bank has a minimum coverage area that includes one local neighbourhood (Ministry of Environment and Forestry Republic of Indonesia, 2021) A neighbourhood is the smallest community unit within a village, typically comprising a minimum of 10 families (Government of Republic of Indonesia, 2014)
Customers	Households (Brilyana, 2023), Unit waste banks (Auliani, 2020)	Households (Ragiliawati and Qomaruddin, 2020) School students (Ekwarso and Fitria, 2015) Traditional market trader (Melyanti, 2014) Office employees (Yustiani, 2019)
Waste buyers	Waste recycling industry (Auliani, 2020) Municipality-level intermediates (*Pelapak Besar*) (Surya, 2021); also known as collectors (*Bandar*) (Damanhuri, 2012) or dealers (Chaerul et al., 2014)	Central waste bank (Ginting et al., 2022) Intermediates (*Pengepul*) (Dewi et al., 2021)
Activities	Types of activities that can be carried out by the central waste bank Customers’ Waste saving (Auliani, 2020; Yayasan Bina Bhakti Lingkungan, 2022) Buying waste from the unit waste bank (Ginting et al., 2022) Buying waste from the small scale individual waste pickers (Suryani, 2014) Providing waste collection service free of charge (Ginting et al., 2022) Providing paid waste collection services (Veronika, 2022) Organic waste processing activities (Wahyuningrum, 2018) Plastic waste processing activities (Adventa et al., 2020) Selling the sorted and collected waste to the waste recycling industry (Auliani, 2020) Conducting waste Management Education for the community (Ardhiansyah and Budihartanti, 2022)	Types of activities that can be carried out by the unit waste bank: Customers’ Waste saving (Saputro et al., 2016) Providing waste collection service free of charge (Dongoran et al., 2018) Composting (Saleh, 2015) processing organic waste into liquid fertilizer (Sianturi, 2021) Selling the sorted and collected waste to the central waste bank (Ginting et al., 2022) Selling the sorted and collected waste to the solid waste buyer (Dewi et al., 2021) Saving waste at the central Waste Bank (Auliani, 2020)

### How does a waste bank work?

**Figure 3.**
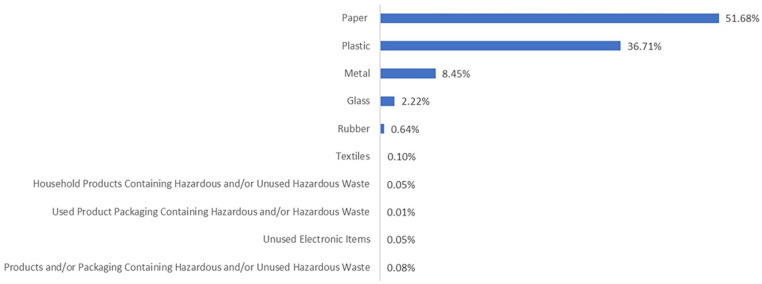
The types of recyclable waste managed by waste banks in general in 2023 (taken from https://simba.menlhk.go.id/portal on 28 January 2024).

Waste bank activities encompass both customer-related activities and those carried out by waste bank administrators. Waste bank customers predominantly comprise individuals as household representatives from the community (Wartama and Nandari, 2020). Customers are responsible for sorting and collecting solid waste from their residence, which they then deliver to the waste bank. Customers are also responsible for the cleanliness of the waste they deliver because waste banks only accept waste in a clean condition. The type of waste must have already been determined by the waste bank itself. Only certain types of recyclable materials that have value in the existing recycling market can be accepted by waste banks including paper, cardboard, plastic bottles, cans, glass and metal. Customers place their sorted waste into containers (Ministry of Environment and Forestry Republic of Indonesia, 2021; Purnama Putra et al., 2018; Raharjo et al., 2017; Ulhasanah, 2018) (Figure 3).

For recyclable wastes that are not accepted by waste banks or cannot be sold in the existing recycling market, they will be classified as residual waste. Generally, residual waste is disposed of in landfills (Paradita, 2018). Some individuals have attempted to process recyclable waste residuals into products such as paving blocks for construction materials (Kader et al., 2021) or other recycling crafts like glass coasters or artificial flowers (Muryani et al., 2020) that can be sold.

Customers take their sorted waste to the waste bank, situated in a designated waste collection location at a predetermined times (Mulyadi and Afandy, 2021). However, there are several waste banks that offer waste collection services to their customers at specific times using vehicles owned by the waste bank. These collection services may be fee-based, as demonstrated by the waste bank ‘*Resik Apik*’ in Pati Regency, Central Java Province (Ahmad, 2019) or offered as a free-of-charge service by waste banks such as the central waste bank ‘*Berseri*’ in Deli Serdang Regency, North Sumatra Province (Ginting et al., 2022) and the waste bank ‘*Mutiara*’ in Medan City, also located in North Sumatra Province (Dongoran et al., 2018).

Upon arrival at the waste bank, the sorted waste is weighed, and the weight is recorded in both the customer’s savings book and the waste bank’s administrative book. Similarly, when customers’ waste is transported using vehicles owned by the waste bank, the segregated waste is unloaded, weighed and recorded in the customer’s savings book and the waste bank’s administrative book. The waste bank manage assigns a price for each type of waste, which is also recorded in the customer’s savings book. The sorted waste, once weighed and recorded, is stored in the waste collection room until it is sold to waste buyers. The waste can be sold either by inviting waste buyers to the waste bank location or by transporting the sorted waste from the waste bank to the waste buyer’s location (Dhewanto et al., 2018; Surya, 2021).

**Figure 4.**
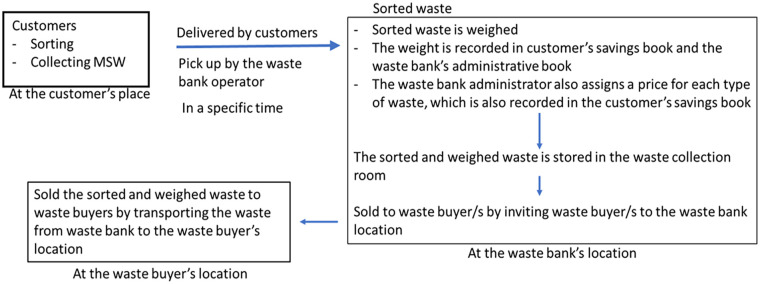
A process flow of a waste bank activities.

Moreover, the amount and types of waste managed by each waste bank may vary depending on the materials that waste buyers are willing to purchase. Generally, there are different types of waste buyers for the waste collected by waste banks. Unit waste banks, due to limited storage space and a relatively small storage capacity, usually sell the waste to central waste banks or intermediaries (Putri et al., 2018). On the other hand, central waste banks have waste buyers such as municipality-level intermediaries or directly engage with the recycling industry (Addinsyah and Warmadewanthi, 2020; Lokahita et al., 2019; Warmadewanthi et al., 2021) (Figure 4).

Intermediaries and municipality-level intermediaries play distinct roles in the informal waste recycling sector (Indrawati, 2011). Intermediaries purchase sorted waste from scavengers, unit waste banks or other sources, and they are responsible for further sorting, washing, drying and packing of the waste. They then sell the sorted and processed waste to municipality-level intermediaries (Damanhuri, 2012). Municipality-level intermediaries specifically purchase sorted and processed waste from intermediaries and central waste banks. They engage in more detailed waste sorting activities, such as sorting beverage bottles based on their colour and material type (Lestari, 2017). Additionally, municipality-level intermediaries also perform pre-treatment activities, including grinding the sorted waste, before selling it to waste recycling plants (Surya, 2021).

The price difference between the price given to the customer for their savings and the price received from the waste buyer is used to finance the operations of the waste bank. Customers commonly can only withdraw their waste savings in cash after a specified period, usually aligned with the Islamic holiday of Eid al-Fitr or the beginning of the new school year (Ekwarso and Fitria, 2015; Setiawan et al., 2021). However, in some locations, such as in ‘*Gaposi Sejahtera*’ waste bank in Mojokerto Regency East Java Province (Ardiantoro and Rohmah, 2019) and ‘*Asoka*’ waste bank in Bekasi West Java Province (Septiani, 2022), waste savings are not converted into cash but are utilized for other purposes, such as paying property and building taxes, electricity bills or health insurance premiums.

In addition, it is likely that there are differences in the application of waste banks in rural and urban areas and this is one of the future research objectives.

### Waste bank: New costumers

New customers express their interest in joining after witnessing the success of a waste bank or through word to mouth communication about the advantages of waste banks (Pujianto et al., 2021; Umyati et al., 2018). New customers may join following waste bank promotional events organized by the managers or in collaboration with waste management education initiatives conducted by local government (Suwerda et al., 2019).

To attract new customers, many waste banks have adopted mobile applications to promote their activities (Bakti et al., 2020; Destriana et al., 2021; Marliana and Firmansyah, 2019; Setyawati et al., 2019). These applications provide information about the waste bank’s locations, services offered and the benefits of becoming a customer. This approach has been implemented by several waste banks, including the ‘Bank Sampah Induk Bantul’ (Wulandari and Alam, 2018) and ‘Gemah Ripah’ waste bank (Handarkho and Irianto, 2016) in Bantul, Special Province of Yogyakarta, as well as the ‘Melati Bersih’ waste bank in Depok, West Java Province (Masruroh et al., 2015).

Furthermore, to attract new customers, many waste banks also have developed non-waste management activities that are highly beneficial to the community, such as savings programmes for waste, which are later converted into various products like gold, household essentials and even health insurance (Khoirunnisa and Sari, 2021; Pratama, 2022; Zulkodri, 2016).

In addition, many studies in Indonesia have shown that there are two main factors influencing a person’s interest in participating in waste banks: the dissemination of information related to waste banks conducted by the government or other actors, such as waste bank administrators and non-governmental organizations (NGOs) (Farasari Nur Bayanana, 2022; Hajar, 2022; Merly Mutiara Saputri et al., 2015; Nurhajati, 2022; Ratiabriani and Purbadharmaja, 2016; Yuliana and Wijayanti, 2019) and real-life examples of economic benefits or other advantages for waste bank customers demonstrated by waste bank applications within their surrounding area (Darmawan et al., 2019; Nurhidayah, 2018; Saputro et al., 2016; Triwardani, 2013; Yuliana and Wijayanti, 2019).

Beside those two factors, there are several other factors that play a role in influencing someone’s interest in becoming a waste bank member, such as family support for active participation in waste banks (Solihin et al., 2019), busyness due to work affecting waste management behaviour at home (Dongoran et al., 2018; Hajar, 2022; Saputro et al., 2016), and the absence of waste separation facilities in households (Yuliana and Wijayanti, 2019).

### Mechanisms for establishing a waste bank

There are no fixed technical guidelines for the establishment of a waste bank. The initiation of a waste bank is typically undertaken by individuals or groups from the community, community-based groups or other entities such as local government agencies, universities and NGOs (Dongoran et al., 2018; Ratnah et al., 2021; Setyoadi, 2018). The lack of adequate government infrastructure for waste collection or insufficient capacity of existing waste management infrastructure to accommodate the rising volume of waste act as triggers for establishing a waste bank (Asri, 2011).

Conversely, the formation of government-sponsored waste banks can be attributed to two factors. The central government has set targets for the establishment of waste banks in Indonesia, as stated in Presidential Regulation No. 97 of 2017. This regulation has been translated into various programmes by many district and municipal governments to develop waste banks in their respective areas. For example, in Jombang Regency, East Java Province, the Regent of Jombang initiated a programme called ‘one village, one waste bank’ (Iftitah and Musta’in, 2018). Similarly, the Yogyakarta City Government in the Special Region of Yogyakarta has a programme called ‘one neighbourhood, one waste bank’ (Dinas Lingkungan Hidup Kota Jogjakarta, 2017). The inclination of local governments, including village governments, to promote business activities through the establishment of waste bank business units, is another driving factor. An example of this is the waste bank business unit managed by the village-owned enterprise of ‘*Setia Asih*’ in Bekasi Regency, West Java Province (Nasution and Agustin, 2020).

To establish a waste bank, the waste bank initiator collaborates with local community leaders to organize meetings that invite potential customers, potential waste bank managers and government/university/NGO representatives as resource persons (Ahmadi et al., 2021; Mahmud and Popoi, 2019; Wardany et al., 2020). Typically, these meetings are held in public meeting halls or at the homes of community leaders (Hidayah et al., 2021). The establishment of a waste bank entails at least four meetings in sequential order, including: (1) boosting the public’s understanding of waste management principles using the 3R principle; (2) presenting the operational techniques of waste banks, which may be supported by visits to established waste banks; (3) reaching a consensus on establishing a waste bank with prospective customers, identifying the waste bank’s location and forming a waste bank management team and (4) setting the operational guidelines for the waste bank (Putra et al., 2019).

Finally, after the agreement is reached on the composition of the waste bank management team, the location of the waste bank and the customer data at the initial phase of waste bank formation are submitted to the village government for the creation of a decree endorsing the establishment of the waste bank, signed by the head of the village (Arifin et al., 2020; Syafrudin et al., 2019).

## Waste bank success in Indonesia

### The role of waste banks in enhancing the quality of life in communities

Functioning waste banks have proven to offer several benefits. Firstly and foremost, requiring customers to sort their waste before depositing it in the waste bank improves waste management (Wulandari, 2014; Wulandari et al., 2017). This practice promotes the proper disposal of waste and reduces the amount of waste that ends up in landfills. Secondly, waste banks indirectly contribute to reducing the improper disposal of waste through littering or burning (Isthofiyani et al., 2016; Kusminah, 2018; Rahmadani, 2020).

Waste banks have the potential to extend the reach of waste management services, particularly in small towns and regencies where local governments have limited funds for waste collection. In Cimahi Regency, West Java Province and Jombang Regency in East Java Province, numerous waste banks have attracted customers from beyond their immediate neighbouring areas. This illustrates the potential for more waste banks to expand the geographical coverage of waste management services where local government is unable to (Iftitah and Musta’in, 2018; Yustiani, 2019).

Waste banks also have the potential to raise income for customers who deposit their waste and to create job opportunities for waste bank managers (Purwanto, 2019; Saputro et al., 2016). Moreover, *Permenlhk 14/2021* is flexible. The activities of waste banks are not limited to waste collection and recycling; they may also manage organic waste (Ministry of Environment and Forestry Republic of Indonesia, 2021).

Interestingly, in many cases, waste banks improvised their activities by doing non-waste management business. Pratama (2022) identifies six types of additional services offered by various waste banks in Indonesia, namely (1) saving waste to obtain gold, (2) saving waste to obtain basic household necessities, (3) saving waste to cover government health insurance premiums, (4) saving waste to pay electricity and water bills, (5) saving waste for children’s education expenses of the waste bank’s customers and (6) saving waste to access savings and loan services. For example, besides offering waste savings programmes, the ‘*Sempu*’ waste bank in Bekasi Regency, West Java Province, also provides loan services to its customers (Khoirunnisa and Sari, 2021). Another example comes from the ‘*Pangkalpinang*’ waste bank in Bangka City, Bangka Belitung Province, where there are alternatives to converting waste bank savings into cash such as internet data packages or household electricity payment packages (Zulkodri, 2016).

These benefits highlight the significant potential for waste banks to promote improved waste management practices and community involvement.

### Potential role of waste banks to increase waste collection areas

**Figure 5.**
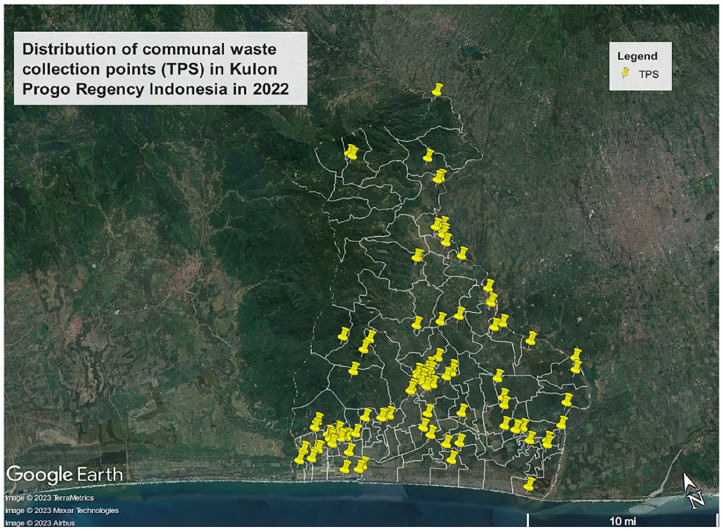
Distribution of communal waste collecting points (*Tempat Penampungan Sementara*/TPS) in Kulon Progo Regency, Special Province of Yogyakarta Indonesia in 2022.

Kulon Progo Regency in Special Province of Yogyakarta, like many other regencies in Indonesia, faces challenges in providing waste collection and transportation infrastructure to each village. Approximately 30% of villages in Kulon Progo Regency lack communal municipal waste collection points (Figure 5). This is especially so for the north western regions and southern coastal regions of Kulon Progo Regency area (Department of Commerce and Industry Government of Kulon Progo Regency Republic of Indonesia, 2023; Department of Public Works Housing and Residential Areas Government of Kulon Progo Regency Republic of Indonesia, 2023). In 2022, it was estimated that 50% of waste was unmanaged (Environmental Department Government of Kulon Progo Regency Republic of Indonesia, 2023).

**Figure 6.**
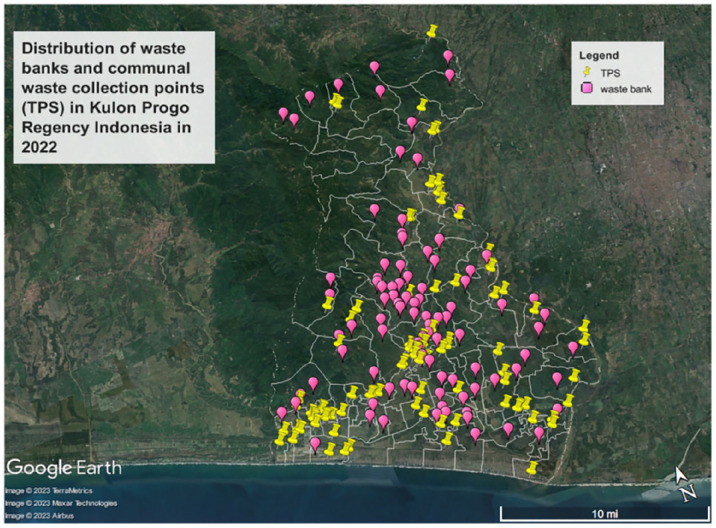
Distribution of communal waste collecting points (*Tempat Penampungan Sementara*/TPS) and waste banks Kulon Progo Regency, Special Province of Yogyakarta Indonesia in 2022.

In 2022, there were 99 waste banks operating in Kulon Progo Regency, covering almost 70% of village (Environmental Department Government of Kulon Progo Regency Republic of Indonesia, 2023). When waste banks were incorporated into the waste management system, the coverage area increased from 65 villages to 77 villages (Department of Commerce and Industry Government of Kulon Progo Regency Republic of Indonesia, 2023; Department of Public Works Housing and Residential Areas Government of Kulon Progo Regency Republic of Indonesia, 2023; Environmental Department Government of Kulon Progo Regency Republic of Indonesia, 2023) representing a 15% expansion in waste service areas (Figure 6). It is worth exploring the potential of expanding the waste bank ideas to all villages in the regency.

## Indonesia’s challenges in implementing waste banks

Despite the successes achieved to date, the implementation of waste banks in Indonesia is accompanied by various challenges.

### Regulatory support for the operational aspects of waste banks

There is currently a lack of specific regulations at the regency level to govern the operational procedures of waste banks. This is important because local characteristics differ and governance arrangements need to accommodate idiosyncrasies (Triana and Sembiring, 2019). A search of 157 regency websites, revealed that only 22 of 157 regencies (less than 15%) have specific regulations for waste banks.

There are exceptions. Gianyar Regency in Bali Province and Demak Regency in Central Java Province have established regulations specifically tailored to managing waste banks. namely Gianyar Regent Regulation Number 80 of 2021 on Guidelines for the Implementation of Household Waste Management and Similar Household Waste (Government of Gianyar Regency Republic of Indonesia, 2021) and Demak Regency Regulation Number 1 of 2023 on Waste Management (Government of Demak Regency Republic of Indonesia, 2023). Their efforts can serve as an example for other regencies to follow.

However, some caution should be taken here. Regency level regulations dedicated to waste banks might not necessarily result in an increase in the number of waste banks in that regency. Table 2 uses four regencies to show the limited correlation between regulations for waste banks and the number of waste banks. Furthermore, investigation is warranted to explore this relationship more deeply.

**Table 2.** Variations in the relationship between the existence of specific regulations on waste banks and the number of waste banks in a regency.

Regency	Existence of specific regulations about waste bank	Number of unit waste banks*	Number of central waste banks*
Mempawah Regency in West Kalimantan Province	No	1	No data
Badung Regency in West Java Province	No	277	No data
Gianyar Regency in Bali Province	Yes	104	3
Pasaman Regency in West Sumatera Province	Yes	4	1

* The data were obtained from the National Waste Management Information System website of the Ministry of Environment and Forestry of the Republic of Indonesia (https://sipsn.menlhk.go.id/sipsn/).

### Lack of infrastructure and limited capacity of waste bank administrators

A significant number of waste bank units do not yet meet the requirements outlined by the Minister of Environment and Forestry Regulation regarding waste bank management guidelines. This includes aspects such as building and equipment specifications, as well as the managerial capabilities of waste bank managers (Cahyadi et al., 2018; Putra et al., 2018). There are several ways that waste banks can resolve these issues that often linked to financial impediments.

The central government has enacted regulations enabling provincial and regency governments to provide grants from regional revenue and expenditure budget (*Anggaran Pendapatan dan Belanja Daerah*) through the Minister of Home Affairs Regulation Number 32 of 2011 (Ministry of Home Affairs of Republic of Indonesia, 2011). If waste banks lack necessary infrastructure, they can seek assistance through their regency or provincial government.

For instance, the ‘*Bestari*’ waste bank in Purbalingga Regency, Central Java Province, received support from the Purbalingga Regency government, in the form of waste collection vehicles. This assistance is utilized by ‘*Bestari*’ waste bank to facilitate waste bank operations, particularly in terms of providing home collection services for customers, thus making it easier for customers to deposit their waste (PemprovJateng, 2020). Similarly, the ‘*Bedeng Berseri*’ waste bank in Yogyakarta received assistance in the form of waste collection carts from the Yogyakarta City Government, as it effectively motivated the residents to create economically valuable handicrafts from solid waste materials (PemkotJogja, 2022).

To enhance their infrastructure, waste banks also can allocate a portion of their operational profits from their own internal funding. For example, the ‘*Resik Apik*’ waste bank in Pati, Central Java Province, established in 2015, initially possessed a limited number of waste collection vehicles; however, it has successfully expanded its fleet of garbage trucks over time to manage the growing waste volume by managing the profits generated from the waste bank activities (Ahmad, 2019).

Alternatively, waste banks can collaborate with NGOs to secure assistance from private entities through extended producers’ responsibility initiatives. A notable case is observed in Klaten Regency, Central Java Province, where several waste banks partnered with the ‘*Lestari*’ NGO obtain financial support from Danone-Aqua, a bottled mineral water producer, aimed at enhancing the waste bank infrastructure (Surya, 2021). This collaboration involving private sector participation in waste bank development has garnered attention from the Ministry of Environment and Forestry of the Republic of Indonesia and has been adopted as a national strategy in reducing plastic waste in Indonesia (Ministry of Environment and Forestry Republic of Indonesia, 2020).

Insufficient managerial skills of waste bank administrators can increase the operational costs of unit waste banks, which can lead to a mismatch between operational costs and earnings. For instance, the ‘*Rangga Mekar*’ waste bank in Bogor City, West Java, experiences monthly losses due its significant fixed operational costs that includes building rent (Elza et al., 2020). These losses could have been anticipated if the administrators of the waste bank possessed better business management skills like find a cheaper building. Another example is provided by Febiola (2021) who explains that many waste bank operators in Malili Regency, South Sulawesi Province, fail to consider the operational costs of waste transportation when selling the waste to recycling industry suppliers. As a result, the income from waste sales is lower than the operational expenses of the waste bank.

To address these concerns, waste bank administrators in some locations have formed networks, such as the Independent Waste Management Network (‘*Jejaring Pengelolaan Sampah Mandiri*’ or JPSM), with the support of the Provincial Government of Yogyakarta Special Region. The primary function of JPSM is to provide managerial and waste management training for waste bank operators, as well as assist in connecting waste banks with parties that can become potential waste buyers (Putra et al., 2021; Setianingrum, 2018; Suwerda et al., 2019).

### The issue of waste banks’ capability to manage the waste management process

The volume of waste managed by waste banks in Indonesia is low compared to waste generation. The Ministry of Environment and Forestry of the Republic of Indonesia reported for 2022 that waste banks processed less than 1% of all Indonesia’s waste. In their study conducted in Bandung City, Wang et al. (2018) concluded that waste banks still have a limited role in the collection and final disposal phases of waste management. On the other hand, some waste banks, especially unit waste banks type like the ‘Desa Kaligerman’ Waste Bank in Lamongan Regency, East Java Province (Chotijah, 2019), the ‘Bersinar’ Waste Bank in Bandung Regency, West Java Province (Sukartono et al., 2023) and the ‘Apik’ Waste Bank in Semarang Regency, Central Java Province (Oktaviana et al., 2022), experience difficulty to maintain their customers. That is because many of their customers prefer to sell their waste to the waste pickers.

Additionally, waste pickers, although often associated with social issues in many areas of Indonesia (Damanhuri and Padmi, 2012), have an important role in the country’s waste management. The waste-picking activities performed by waste pickers are individual activities, involving approximately 15,000 people and are conducted in both residential areas and landfills. According to the Indonesia Ministry of Environment and Forestry in 2023 around 83,000 tonnes of waste are recycled through waste pickers activities. The role of these waste pickers is quite significant, considering the high amount of unmanaged waste in Indonesia, reaching around 24 million tonnes in 2023 (approximately 35% of the total waste generated). Therefore, all efforts by various stakeholders, including waste pickers, to reduce the amount of unmanaged waste are essential for waste management in Indonesia.

However, waste pickers remain unregulated in Indonesia’s waste management policies, both at the central and local levels (provincials, cities and regencies/small cities). This contrasts with waste banks, whose activities are already governed by the central government through regulations from the Ministry of Environment and Forestry. In some regencies, such as Gianyar Regency in Bali Province and Demak Regency in Central Java Province, specific regulations related to waste bank activities have been issued. Nevertheless, the non-regulated side of waste management, such as waste pickers, is an area worthy of closer inspection – specifically to investigate if there is a tension between the role of waste banks and waste pickers. However, it is important to note that this research only focuses on the regulations and regulated waste services.

In addition, the definition of the informal and formal sectors in waste management in Indonesia remains unclear, especially concerning waste banks. This is because, even though waste banks are regulated by the central government through the Ministry of Environment and Forestry of the Republic of Indonesia Regulation No. 14 of 2021, they have not yet been integrated into the MSW management system in cities or regencies/small cities in Indonesia (Raharjo et al., 2019). Integration would mean that if the government decides to integrate sectors like waste banks, it is not sufficient to mention it in the regulation alone (Sembiring and Nitivattananon, 2010). The government must also consider all the consequences of this decision, including allocating a regular operational budget for waste banks and providing necessary facilities for them. This gap presents an interesting area for future research.

Increasing the volume of waste managed by waste banks can be achieved by creating more waste banks in places where there is no local government waste collection services, increasing the number of bank customers and encouraging customers to increase the amount of waste deposited (Putri et al., 2018). There have been at least three efforts made by waste banks to attract new customers. Firstly, the waste bank administrators can conduct education campaigns to raise awareness among the community about the role of waste management through waste banks. This is regularly carried out by the ‘*Rumah Harum*’ waste bank in Depok City, West Java (Ardhiansyah and Budihartanti, 2022). Alternatively, waste bank administrators invite waste management practitioners from waste bank associations such as *ASOBSI* (‘*Asosiasi Bank Sampah Indonesia*’ or Indonesian Waste Banks Association) and *PERBANUSA* (‘*Perkumpulan Pengelola Sampah dan Bank Sampah Nusantara*’ or Nusantara Waste Banks Association) to provide additional information to the public regarding waste management (Mulyantini and Irawatie, 2023; Nugraha et al., 2023).

Secondly is using mobile-based information technology to facilitate waste collection scheduling and promote waste bank activities. This has been implemented by ‘*Malang*’ waste bank in Malang City, East Java Province (Wardhana et al., 2019), and ‘*Vila Dago*’ waste bank in South Tangerang Regency, Banten Province (Sansprayada and Mariskhana, 2020), which have shown positive outcomes in increasing community participation in waste bank savings activities.

Thirdly, it involves offering waste purchase prices that are nearly the same or even better than what is offered by waste pickers, as demonstrated by the Malang Waste Bank in the city of Malang, East Java Province (Suryani, 2014), as well as providing various benefits to the customers, ranging from internet data package payments to microfinance opportunities (Khoirunnisa and Sari, 2021; Zulkodri, 2016)

## Conclusion

Waste banks, initially developed by a local community in Bantul Regency, Special Province of Yogyakarta, have rapidly expanded throughout Indonesia, making them the most prevalent form of community-based solid waste management in the country. This widespread adoption suggests there is potential for waste banks to address the issue of unmanaged MSW. They actively promote proper waste disposal practices, effectively reducing the amount of waste that ends up in landfills and contributing to a cleaner environment. Waste banks not only contribute to reducing unmanaged waste but may also have positive impacts on the local economies, creating opportunities for increased income and offering additional services to communities including banking opportunities, savings and loans and the convenience of purchasing daily necessities.

In Indonesia, waste banks already play a significant role in improving the quality of life for some communities. Additionally, to ensure the continued development and success of waste banks, it is crucial to garner support from various stakeholders. This support should encompass providing adequate infrastructure and capacity building for waste bank administrators, ensuring their operations are sustainable in the long run. Although there are regulatory opportunities to support waste bank development, further investigation is needed to understand the specific effects of regulations at the regency level on waste bank growth in different regions. Therefore, it is essential to review and customize support mechanisms for waste banks based on local characteristics, enabling them to maximize their potential and make a significant impact in their respective communities.
